# Sensory processing and child appetitive traits: findings from the ROLO longitudinal birth cohort study

**DOI:** 10.1186/s12937-024-01040-1

**Published:** 2024-11-01

**Authors:** Anna Delahunt, Sophie Callanan, Eileen C. O’ Brien, Aisling A. Geraghty, Sharleen L. O’ Reilly, Ciara M. McDonnell, Emma Hokey, Fionnuala M. McAuliffe

**Affiliations:** 1 UCD Perinatal Research Centre, School of Medicine, University College Dublin, The National Maternity Hospital, Dublin 2, Ireland; 2 School of Biological and Health Sciences, Technological University Dublin, Dublin, Ireland; 3 School of Agriculture and Food Science, University College Dublin, Dublin 4, Ireland; 4 Department of Paediatric Endocrinology & Diabetes, Children’s Health Ireland Temple St & Tallaght, Dublin, Ireland; 5 Discipline of Paediatrics, School of Medicine, Trinity Research in Childhood Centre (TRICC), Trinity College, Dublin, Ireland

**Keywords:** Sensory profile, Oral sensory processing, Appetitive traits, Child, Maternal

## Abstract

**Background:**

Oral sensory hypersensitivity has been linked with fussy eating predominantly in non-typically developing children. We hypothesized that child ‘Oral’ (touch, smell and taste in the mouth) and ‘Social-Emotional’ (response to social expectations) sensory processing are associated with child appetitive traits in typically developing preteen children. Additionally, we explored relationships between maternal sensory profiles and their offspring’s sensory profile.

**Methods:**

This is secondary analysis of 130 mother-child dyads from the 9–11-year-old follow-up of the ROLO longitudinal birth cohort study. The Dunn Sensory Profile (Adolescent/Adult) and the Dunn Child Sensory Profile 2 were used to assess maternal and child sensory profiles, respectively. The Children’s Eating Behaviour Questionnaire was used to assess child appetitive traits. Multiple linear regression examined associations between ‘Oral’ and ‘Social-Emotional’ segments of the child sensory profile and child appetitive traits. Associations between maternal and child sensory profiles were examined using sensory profile quadrants (Dunn’s sensory processing framework). These refer to four distinct patterns of sensory processing that indicate how an individual responds to sensory input.

**Results:**

In total, 130 mother-child dyads were included. In adjusted analysis child ‘Oral’ sensory processing was associated with higher mean scores in the appetitive traits ‘Desire to Drink’ (B = 0.044, 95% CI = 0.025,0.062) and ‘Food Fussiness’ (B = 0.080, 95% CI = 0.059,0.101) and with lower mean scores in ‘Enjoyment of Food’ (B=-0.038, 95% CI -0.055,-0.022). Child ‘Social-Emotional’ responses were associated with higher mean scores in ‘Desire to Drink’ and ‘Food Fussiness’. Higher scores in the maternal sensory profile quadrants of ‘Sensitivity’, ‘Avoiding’ and ‘Registration’ were positively associated with higher scores in the corresponding child sensory profile quadrants.

**Conclusion:**

Our findings suggest that in typically developing children presenting with fussy eating, oral sensory hypersensitivity and higher scores in social-emotional responses to sensory processing may be an underlying determinant. The relationship observed between ‘Oral’ and ‘Social-Emotional’ sensory processing segments and ‘Desire to Drink’ requires further research to ascertain the type of drinks being consumed and how this impacts appetite. Further research is also required to explore the influence of maternal sensory profile on their offsprings response to sensory input.

**Supplementary Information:**

The online version contains supplementary material available at 10.1186/s12937-024-01040-1.

## Background

Feeding is a sensorimotor skill that evolves during the first years of life. It is a highly complex process and is dependent on learning from experience and neurological maturation. Sensory processing is the ability to process and interpret information from our senses (e.g. vision, touch, taste, body position, hearing). As humans we are surrounded by the sensory events of daily life. Individuals vary in the degree to which they are sensitive to sensory stimuli and how much they enjoy or react to different sensory experiences [1]. How we notice, recognize, interpret and respond to various stimuli determines an individual’s sensory profile. The Dunn [2] sensory processing framework proposes four basic patterns of sensory processing based on an individual’s neurological threshold for detecting and responding to sensory stimuli. These include; ‘Seeking’ which measures the degree of which an individual will seek sensory input, ‘Avoiding’ which measures how much an individual is bothered by sensory input, ‘Sensitivity’ measures an individual’s ability to detect or notice sensation and ‘Registration’ measures the degree to which an individual misses sensory input [2, 3].

The ‘Oral’ sensory processing segment of the Child Sensory Profile 2 (CSP2) measures a child’s response to touch, smell and taste in the mouth [3]. Heightened reaction to smell, touch and taste has been shown to be related to food acceptance, food refusal and food fussiness [4, 5]. In general, when compared with typically developing children, children with autism spectrum disorder (ASD) have been reported to present with heightened levels of oral hypersensitivity [6–8] and higher levels of feeding difficulties [9, 10]. In the typically developing child, fewer studies have examined the impact of oral sensory processing on a range of appetitive traits (dispositions towards foods that differ between individuals [11]).

Fussy eating has been defined as the consumption of a limited variety or quantity of foods through the rejection of a significant amount of familiar and unfamiliar foods [12, 13]. Poor diet quality [14] and anxiety and stress for the family and child [15] are some of the implications of food fussiness. Identifying the underlying factors driving fussy eating behaviors could help direct strategies to address and manage it. Issues with the perceived sensory properties of food may be an underlying factor for rejecting food in both typically and atypically developing children [1, 16]. As foods differ in their sensory properties, the integration of most of the senses are involved in eating. Research has shown that children with aversive reactions to textures, smells and temperature of food show high levels of food fussiness and reluctance to try new foods [17–19]. A recent study of 589 Spanish children aged 3–7 years old observed that taste and smell sensory reactivity was significantly correlated with food selectivity [20]. Refusal to eat fruit and vegetables, and/or unfamiliar foods in childhood has been associated with increased levels of taste, touch and smell sensitivity [21], however, other determinants such as lack of exposure, cultural or parental feeding practices have also been shown to be influential [22, 23].

Appetitive traits are related to both diet quality and child weight status [24–26]. The preteen years are a crucial period for consolidating eating behaviours developed in the early years [14, 27, 28]. Within these years children are developing autonomy in their eating habits, and are often eating outside the home. Prolonged sub-optimal eating habits can significantly impact growth, nutritional status and cognitive development [13, 29–31]. Research has shown that diet quality decreases as age increases [14], therefore it is vital to address barriers to healthy eating habits before they become entrenched.

Twin studies have shown that appetitive traits are developed by a combination of genetics and environmental factors [32, 33], albeit the influence of each differ as per behavior. A twin study of children aged 3.5 years old indicated that food fussiness and fruit and vegetable liking shared common genetic factors [33]. Sensory sensitivity is believed to be an inherent characteristic [34].

Research on the influence of maternal sensory profile on their offspring’s sensory profile is lacking. Few studies have examined if a relationship exists between maternal and child sensory processing patterns and how this may relate to their offspring’s eating behaviors. Research suggests that maternal eating behavior is associated with child eating behavior [35, 36], and that children often imitate the attitudes and behavior of their parents, particularly their mothers [37]. Awareness of how maternal sensory processing patterns influence their offspring’s sensory processing patterns may help provide better understanding of their behaviors.

Food avoidant appetitive traits such as fussy eating have been linked with anxiety and low mood [38, 39] and to higher levels of parent reported negative affect in children [40]. Child temperament has been recognized as a significant factor in how a child behaves in the feeding environment [41]. The ‘Social-Emotional’ responses associated with sensory processing segment of the CSP2 measures a child’s expressiveness in a social situation [3] and includes items about self-esteem, emotions and fears. To date, research has not explored the relationship between child ‘Oral’ hypersensitivity and ’Social-Emotional’ responses associated with sensory processing, and how this relates to appetitive traits. Cross sectional and longitudinal studies have observed a link between childhood fussy eating and the child with a predisposition to get upset or distressed easily (the emotional temperament) [42–44]. Zucker, Copeland [39] observed that food fussiness was associated with current and future psychopathological symptoms (anxiety, depression, attention-deficit disorder). A deeper understanding of the relationship between fussy eating and heightened ‘social-emotional’ sensory processing argues for the value of early identification of predictors of fussy eating, and how this may help when planning interventional strategies.

In the current study, we describe the sensory profiles of a cohort of healthy mother-child pairs. Our primary aim explored the relationship between child ‘Oral’ sensory processing and child appetitive traits at 9–11 years old in typically developing children. A secondary aim explored associations between child ‘Social-Emotional’ responses associated with sensory processing and child appetitive traits. Lastly, we explored relationships between maternal and child sensory profile quadrants as per the Dunn’s Sensory Processing Framework [2].

## Methods

### Study population

This is secondary cross-sectional analysis of 130 mother-child pairs from the ROLO (Randomized controlled trial of a low glycemic index diet) longitudinal birth cohort study. The primary study took place in the National Maternity Hospital, Dublin, Ireland from 2007 to 2011, where 800 women who were on their second pregnancy, and had previously given birth to an infant weighing over 4 kg (macrosomia) were recruited. Detailed methods and results of the primary study have been published elsewhere [45]. Women and their second born child (*n* = 759) have been followed up at 3 and 6 months, 2 years, 5 years and at 9–11 years (Preteen study) as part of the ROLO longitudinal birth cohort study. Data for the current study is from the 9–11-year-old (Preteen study) follow-up. Mother-child pairs were invited to attend a study day when their child turned 9 years old. In all, 432 mother-child pairs participated. Of the 432 ROLO Preteen follow-up participants, 132 completed maternal and child sensory profiles and the Children’s Eating Behaviour Questionaries. Two children had a diagnosis of ASD and were therefore excluded, leaving a study cohort of 130 participants.

### Ethical Approval

Ethical approval for the 9 to 11-year follow-up study was granted by University College Dublin (UCD) Research Ethics Committee (LS-15-06-Geraghty-McAuliffe). Details on retention strategies implemented to maintain study engagement in the ROLO cohort were published previously [46].

### Maternal baseline data, early child feeding and general health of children at 9–11 years old

Baseline maternal data were collected during pregnancy on maternal age at delivery, education level and ethnicity. Women choose between six categories ranging from no schooling to third level or above education. Maternal ethnicity was dichotomized into white Irish or other.

Breastfeeding exposure and duration were recorded at 6 months, 2 years and 5 years. At each of these timepoints, mothers reported if they had ‘breastfed ever’ and the duration (weeks) that they had breastfed for. As a marker of any breastfeeding exposure, a composite variable was created to categorize participants into ‘breastfed ever’ or ‘never breastfed’. At the 2 year and 5-year follow-up mothers reported the age (weeks) their infant had commenced complimentary feeding. This data was dichotomized into whether the child had started complimentary feeding as per European [47] and national [48] recommendations or not, that is that complementary foods are not introduced before four months (17 weeks), but should not be delayed beyond six months (26 weeks). Lifestyle questionnaires were completed by mothers about the general health of their child during the 9–11-year-old follow-up study. History of ASD, sensory processing disorder, ADHD or any other physical or developmental diagnoses were recorded.

### Maternal and child anthropometry at 9–11-year follow-up

All measurements were obtained and calculated by a trained researcher. At the 9–11-year-old visits, maternal and child weight were measured using a calibrated stand-on digital weighing scale (SECA 813 GmbH & co. Kg. Hamburg, Germany ) to the nearest 0.1 kg. Participants were measured in light clothing without shoes. Mothers and child’s standing height was measured, without shoes, with head aligned in the Frankfort plain, using a free-standing stadiometer (SECA 217 GmbH & co. Kg. Hamburg, Germany) and measurements were recorded to the nearest 0.1 cm. Body mass index (BMI) was calculated for mother and child as kilogram per square meter (kg/m^2^). Maternal BMI was classified as per the World Health Organization classification system [49]. Children’s BMI scores were converted to standardized z-scores according to the 1990 UK age-and sex-specific reference data using Excel LMS Growth macro [50]. BMI z-scores were categorized using the World Health Organization criteria for children aged 5 to 19 years [51].

### Exposure: Sensory Profiles

The Dunn 60-item Adolescent/Adult Sensory Profile (A/ASP) [52] was used to assess maternal sensory profiles and the Dunn 86-item Child Sensory Profile 2 (CSP2) caregivers questionnaire (for children aged 3–14 years old) was used to assess child sensory profiles [3]. The A/ASP Sensory Profile and the CSP2 evaluate an individual’s behavioral responses to everyday sensory experiences. Scores are generated to provide an individualized profile of sensory processing across four quadrants. The Dunn four quadrant model of sensory processing is based on two domains; neurological threshold and behavioral response. The neurological construct refers to the threshold of an individual’s response to sensory stimuli and can range from low to high. The second construct, behavioral response, is based on whether an individual has a passive or active strategy in how they respond to their environment. These two constructs cross to form four major sections. The four quadrants are similar in the A/ASP and the CSP2 albeit with minor variations in their titles and differences in the statements within the profiles. The quadrants are (CSP2); Sensory Seeking (Seeking/Seeker) – a high score indicates the individual craves high amounts of environmental stimuli *(e.g. craves certain foods*,* tastes or smells’)* (CSP2 oral section), Sensation Avoiding (Avoid/Avoider) - a high score indicates avoidance of high levels of stimuli and being upset by high sensory input (*e.g. I move away when others get too close to me’)* (A/ASP Touch section); Sensory Sensitivity (Sensitivity/Sensor) – high scores indicate those who are sensitive to stimuli and quickly detect it in their environments *(e.g. ‘limits self to certain food textures’)* (CSP2 Oral section) and Low Registration (Registration/Bystander) individuals with high scores typically miss more sensory input than others *(e.g. I don’t smell things that other people smell’)* (A/ASP Taste/Smell section). Quadrant scores are calculated from the raw scores obtained from the statements within the sensory profiles via a 5-point Likert scale ranging from 1 to 5 (1 (Almost Never) to 5 (Almost Always). The A/ASP raw scores are derived from six sensory processing subscales; taste/smell, movement, visual, touch, activity level and auditory processing. The CSP2 raw scores are derived from nine sensory processing subscales; auditory, visual, touch, movement, body position, oral sensory processing and three subscales that reflect emotional and behavioral responses that might indicate a child’s sensory processing abilities; conduct, social-emotional responses (items indicate the child’s psychosocial coping strategies) and attentional responses associated with sensory processing. Quadrant scores are placed on pattern grids that visually represent how the individual’s sensory processing is compared to that of other adolescents/adults or children.

The CSP2 was completed by the child’s mother to evaluate the child’s behavioral response to everyday sensory experiences. The two CSP2 subscales used as predictors of appetitive traits were ‘Oral’ sensory processing’ and ‘Social-Emotional’ responses associated with sensory processing. The ‘Oral’ sensory processing segment (10 items) measures the child’s response to touch, smell and taste in the mouth *(e.g. gags easily from certain food textures or food utensils in the mouth)*. The ‘Social-Emotional’ responses associated with sensory processing segment (14 items) measures the child’s expressiveness in a social situation *(e.g. Is sensitive to criticisms)*.

Mothers were posted the adult and child sensory profiles with instructions provided on how to complete both profiles. Completed profiles were returned either at in-person study visits or by post using the stamped addressed envelope provided. Participants were instructed to answer questions regarding how they generally respond to sensations, as opposed to at that specific time. Instructions were provided to complete the questionaries reflecting pre-COVID-19 sensory status. The adult and child sensory profiles have good internal and external validity [34]. During development the internal consistency (Cronbach alpha) method was used to estimate the reliability of the A/ASP and the CSP2. For our cohort, Cronbach alpha for the A/ASP quadrants ranged from 0.70 to 0.78. The Cronbach alpha for CSP2 quadrants ranged from 0.840 to 0.941 and for the oral sensory processing and social-emotional processing segments were 0.884 and 0.941 respectively.

### Outcome: Child appetitive traits

The Children’s Eating Behaviour Questionnaire (CEBQ) [53] was completed by mothers to evaluate their child’s appetitive traits. This is a 35-item psychometric tool, which was designed to assess appetitive traits in children. The CEBQ has been shown to have good internal and external reliability [54, 55] and has been validated against observational measures of eating behavior, corresponding well to children’s energy intake [54]. Each item response is graded on a 5-point Likert scale (‘never to always’) with 5 items within the CEBQ being reverse scored, due to negative phrasing. Each question relates to one of eight eating styles which can be classed as either ‘Food approach’ or ‘Food avoidant’. The food approach category includes ‘Food Responsiveness’, ‘Enjoyment of Food’, ‘Emotional Overeating’ and ‘Desire to Drink’. The ‘Food Responsiveness’ subscale contains five questions which assess a child’s appetite and whether they display a heightened response to external food cues. The ‘Enjoyment of Food’ subscale consists of four questions assessing a child’s enjoyment and interest in food. The ‘Emotional Overeating’ subscale contains four questions, which explore overeating as a reaction to negative emotions such as annoyance, worry, anxiety or boredom. The ‘Desire to Drink’ subscale contains three questions assessing an increased desire for frequent beverage consumption. The ‘Food avoidant’ category includes ‘Satiety Responsiveness’, 'Slowness Eating’, ‘Emotional Undereating’ and ‘Food Fussiness’. The ‘Satiety Responsiveness’ subscale consists of five questions, exploring a child’s inability to respond to internal satiety cues and reduction in intake due to perceived fullness. ‘Slowness Eating’ comprises of four questions pertaining to the length of time a child takes to finish their meal, indicating a lack of interest in eating. ‘Emotional Undereating’ is assessed by four questions, exploring a child’s inclination to limit food intake in times of negative and positive emotions, such as being upset, sad, happy or tired. The ‘Food Fussiness’ subscale includes six questions that assess food avoidance, selectivity and a lack of interest in food. Each subscale is summed to give a total score and this is divided by the number of items within the subscale to give a mean score of the sum. A higher score indicates the child is more likely to express this eating behavior. In the current sample Cronbach’s alpha for the CEBQ ranged from 0.699 to 0.917, thus all questions were included in the analysis.

### Statistical analysis

Continuous data were tested for normality using the Kolmogorov–Smirnov test and visual inspection of histograms. Normally distributed variables were reported as mean and standard deviation (SD). Non-parametric variables were reported as median and interquartile range (IQR 25th -75th ). Categorical variables were reported as n (%). Appetitive trait variables were normally distributed and parametric tests were used. Cronbach α was performed on each subscale of the CEBQ, A/ASP and the CSP2 to assess the internal consistency of each in our cohort. As part of our descriptive analysis, child characteristics, child sensory profile scores and child appetitive traits were examined across child sex using Independent sample t-tests for normally distributed variables or Mann Whitney U tests for non-normally distributed variables. Spearman’s correlations explored relationships between child ‘Oral’ and ‘Social-Emotional’  sensory profile segments and child appetitive traits. Spearman’s correlations explored relationships between maternal and child sensory processing quadrants. In both cases, where a moderate to strong correlation was observed, further analysis was conducted using linear regression. Adjusted and non-adjusted linear regression examined associations between child ‘Oral’ and child ‘Social-Emotional’ sensory profile segments (predictors) and child appetitive traits (dependent variables). Prior to regression analysis, normality, homoscedasticity, and linearity were assessed. A forced entry approach was used for the addition of confounders to multiple regression models. Potential confounders were decided upon a priori based on the literature and on author consensus. These included child age, child sex, breastfeeding exposure, whether the child met timing recommendations for the introduction of complementary feeding or not, maternal education level, original RCT group, maternal and child BMI at the 9–11-year follow-up. To address similarity in one item contained in the child ‘Oral’ sensory processing segment *(‘rejects certain tastes or food smells that are typically part of children’s diets’)* and two items in the CEBQ ‘Food Fussiness’ domain (‘My child enjoys tasting new foods’ (reverse scored) and ‘My child enjoys a wide variety of food’ reverse scored), post hoc sensitivity analysis was carried out by excluding the overlapping content in the sensory profile. This yielded similar associations to previous analyses. In the multiple linear regression analysis examining associations between maternal and child sensory processing quadrants, maternal education was not included as a confounder in the regression models. To account for multiple testing, the Benjamini-Hochberg correction method was applied with a false discovery rate of 0.05. In total, 54 comparisons were completed within the analyses. This research was exploratory in design; thus, power and sample size calculations were not included for this secondary analysis. Statistical analyses were completed using IBM Statistical Package for Social Sciences (SPSS) for Windows, version 27.0. Armonk, NY: IBM, Corp.

## Results

### Maternal and child characteristics

General characteristics of the cohort (*n* = 130) are presented in Table 1. In the full cohort (*n* = 132), two children had a diagnosis of ASD and no children had a diagnosis of ADHD or a Sensory Processing Disorder. Our analysis excludes the two children with ASD.

### Maternal sensory profiles

The majority of mothers fell within the ‘similar to others’ range for each of the sensory profile quadrants (Additional file 1). Median raw scores (25th -75th) for maternal sensory profile quadrants are presented in Table 1.

### Child sensory profiles and child appetitive traits

The majority of children fell within the ‘similar to others’ range for each of the four sensory profile quadrants (Additional file 2). For the ‘Oral’ sensory processing segment, 13.8% of children had scores of ‘more than’ or ‘much more than others’. Within the ‘Social-Emotional’ sensory processing segment, 16.9% of children scored as ‘more than’ or ‘much more than others’ (Additional file 3). Median (25th -75th ) and mean (SD) scores for child sensory quadrants and sensory profile scores are presented in Table 1. The mean (SD) of the sum of each CEBQ subscale are presented in Table 1. In this cohort, 35% (*n* = 45) of children had a total score (sum of mean) of 4 or more in the CEBQ ‘Food Fussiness’ subscale, indicating that they either ‘often’ or ‘always’ expressed an issue with food acceptance.

**Table 1:** Descriptives of maternal and child characteristics from the ROLO 9–11-year follow-up

	*n* (%)	Mean (median)	SD (IQR)
**Maternal characteristics at 9–11 year old follow-up**			
Age at 9–11-year follow-up (years)	130	(43.3)	(40.6,46.0)
RCT group (intervention), n (%)	73 (55.3)	-	-
Education; completed 3rd level or above, n (%)	85 (70.8)	-	-
Ethnicity (white, Irish), n (%)	118 (90.1)	-	-
BMI (kg/m^2^)	130	(25.56)	(23.26, 28.88)
BMI category ƚ (overweight/obesity), n (%)	72 (56.2)	-	-
**Maternal sensory profile quadrants**			
Low registration (Similar to most = 24–35)	130	28.7	6.7
Sensation seeking (Similar to most = 43–56)	130	47.7	6.9
Sensory sensitivity (Similar to most = 26–41)	130	34.0	8.2
Sensation avoiding (Similar to most = 27–41)	130	33.0	7.8
**Child characteristics at 9–11 year old follow-up**			
Child age (years)	130	(9.8)	(9.3,10.3)
Male, n (%)	68 (51.5)	-	-
Breastfeeding exposure (weeks)	85 (70.8)	-	-
Age of solids introduction (weeks)	118	(26.0)	(21.2,26.0)
Pubic hair distribution (Tanner Stage I), n (%)	103 (88.8)	-	-
Girls breast development (Tanner Stage I), n (%)	50 (84.7)	-	-
BMI (kg/m^2^)	130	(16.85)	(15.54,19.47)
BMI z-score	130	0.3	1.1
BMI z-score category ϯ (overweight/obese) n (%)	37 (28.7)	-	-
**Child sensory profile quadrants**			
Registration/Bystander (just like majority = 20–47)	130	(29.0)	(24.0,34.0)
Seeking/Seeker (just like majority = 21–46)	130	(27.5)	(22.0,35.0)
Sensitivity/ Sensor (just like majority = 18–42)	130	(27.0)	(22.0,33.0)
Avoiding/Avoider (just like majority = 19–43)	130	(30.0)	(23.0,36.3)
**Child sensory profile segments**			
Visual (just like majority = 9–17)	130	11.6	4.0
Body position (just like majority = 5–15)	130	(8.5)	(8.0,11.0)
Oral (just like majority = 8–24)	130	(14.0)	(11.0,20.0)
Social-emotional (just like majority = 13–31)	130	(20.0)	(16.0,27.0)
**Child appetitive traits at 9-11yrs**			
Food Responsiveness	130	2.5	0.8
Emotional Overeating	130	2.0	0.6
Enjoyment of Food	130	3.9	0.7
Desire to Drink	130	2.4	0.8
Satiety Responsiveness	130	2.8	0.7
Slowness eating	130	2.6	0.8
Emotional Undereating	130	2.5	0.7
Food Fussiness	130	2.9	1.0

Mean and standard deviation (SD) presented for normally distributed variables, Median and 25th to 75th Interquartile range (IQR) presented for non-normally distributed variables. Categorical variables presented as n (%.); ϯ WHO BMI classification; ϯ WHO cut-offs for BMI z-scores for children aged 5–19 years old; Mean and SD of CEBQ subscales are derived from the sum of subscale divided by number of items within the subscale; Pubertal status defined as being in Stage I (pre-adolescent) of pubic hair distribution for males; Stage I of pubic hair distribution and Stage I of breast development for females according to the 5-stage Tanner criteria

Abbreviations: ROLO; Randomized cOntrol Trial of LOw Glycaemic Diet

### Differences in child characteristics, sensory processing and appetitive traits across sex

Following adjustment for multiple testing, boys had higher mean (SD) BMI z-score than girls (0.5 (1.2) vs. 0.1 (1.1)) (Table 2). Boys had higher mean (SD) scores for ‘Emotional Overeating’ (2.1 (0.6) vs. 1.9 (0.5)) than girls (Table 2). Girls had higher mean (SD) scores for ‘Satiety Responsiveness’ (2.9 (0.7) vs. 2.6 (0.7) compared to boys. Boys had higher median (25th -75th ) scores in the ‘Sensitivity/Sensor’ quadrant (29.0 (24.0,36.0) vs. 25.0 (21.0,31.8)) compared to girls (Table 2).

**Table 2:** Differences in child characteristics, appetitive traits and segments of child sensory profile across sex

	Boys			Girls			
	*n*	Mean (median)	SD (IQR)	*n*	Mean (median)	SD (IQR)	
**Child characteristics**							
Child age (years)	66	(9.9)	(9.4,10.3)	64	(10.0)	(9.3,10.3)	
Breastfeeding duration ϯ (weeks)	38	(17.3)	(8.0,43.3)	40	(28.2)	(9.0,52.0)	
Age of solids introduction ϯ (weeks)	58	(21.7)	(19.1,26.0)	60	(26.0)	(21.7,26.0)	
BMI z-score	66	0.5	1.2	64	0.1	1.1	*
**Child Appetitive traits**							
Food Responsiveness	66	2.6	0.8	64	2.3	0.8	
Emotional Overeating	66	2.1	0.6	64	1.9	0.5	*
Enjoyment of Food	66	3.9	0.8	64	3.9	0.7	
Desire to Drink	66	2.5	0.8	64	2.3	0.8	
Satiety Responsiveness	66	2.6	0.7	64	2.9	0.7	*
Slowness Eating	66	2.5	0.9	64	2.8	0.8	
Emotional Undereating	66	2.6	0.7	64	2.5	0.7	
Food Fussiness	66	3.0	1.1	64	2.9	0.9	
**Child sensory profile quadrants**							
Registration/Bystander ϯ	66	(29.5)	(24.8,34.0)	64	(27.0)	(23.0,34.0)	
Seeking/Seeker ϯ	66	(28.0)	(22.8,36.3)	64	(27.0)	(22.0,34.0)	
Sensitivity/ Sensor ϯ	66	(29.0)	(24.0,36.0)	64	(25.0)	(21.0,31.8)	*
Avoiding/Avoider ϯ	66	(31.0)	(24.0,42.0)	64	(29.0)	(23.0,33.8)	
**Child sensory profile segments**							
Visual	66	11.7	4.3	64	11.6	3.6	
Body position ϯ	66	(9.0)	(8.0,11.5)	64	(8.0)	(8.0,11.0)	
Oral ϯ	66	(14.0)	(11.0,21.5)	64	(13.0)	(11.0,17.0)	
Social-Emotional ϯ	66	(21.0)	(17.0,30.8)	64	(19.0)	(16.0,24.8)	

Independent samples t test for normally distributed variables or ϯ Mann Whitney U test for non-normally distributed variables were used to explore differences in child characteristics, child appetitive traits and sections of child sensory profiles across child sex. SD Standard deviation; IQR Interquartile range (25th -75th ); CI Confidence Interval

Benjamini-Hochberg False Discovery Rate adjustment applied. *Q = 0.05, **Q = 0.01, ***Q = 0.001

### Correlations between child ‘Oral’ and ‘Social-Emotional’ sensory processing segments and child appetitive traits

Results are presented in Additional file 4. Following correction for multiple testing a positive relationship was observed between child ‘Oral’ sensory processing and ‘Desire to Drink’ (*r* = 0.44), ‘Satiety Responsiveness’ (*r* = 0.24) and ‘Food Fussiness’ (*r* = 0.53). A negative relationship was observed between child ‘Oral’ sensory processing and ‘Enjoyment of Food’ (*r* = -0.32). A positive relationship was observed between the ‘Social-Emotional’ sensory processing segment and ‘Food Responsiveness’ (r = 0.23), ‘Emotional Overeating’ (r = 0.28), ‘Desire to Drink’ (r = 0.44), ‘Emotional Undereating’ (r = 0.24) and Food Fussiness (r = 0.29). Child ‘Oral’ and ‘Social-Emotional’ sensory profile segments were strongly correlated (r = 0.49) (Additional file 5).

### Multiple linear regression - child ‘Oral’ sensory processing and child appetitive traits

In adjusted analysis, and following correction for multiple testing, a positive association was observed between child ‘Oral’ sensory processing and ‘Desire to Drink’ (B = 0.044, 95% CI = 0.025,0.062) and ‘Food Fussiness’ (B = 0.080, 95% CI = 0.059,0.101). ‘Oral’ sensory processing accounted for 35% of the variance of ‘Food Fussiness’ explained by the model. A negative association was observed between child ‘Oral’ sensory processing and ‘Enjoyment of Food’ (B =-0.038, 95% CI = -0.055,-0.022).

### Multiple linear regression- child ‘Social-Emotional’ sensory processing segment and child appetitive traits

In adjusted analysis ‘Social-Emotional’ responses associated with sensory processing were positively associated with ‘Desire to Drink’ (B = 0.023, 95% CI = 0.008,0.038), ‘Emotional Undereating’ (B = 0.018, 95% CI 0.004,0.031) and ‘Food Fussiness’ (B = 0.029, 95% CI = 0.009,0.049). Adjusted and unadjusted results are presented in Table 3.

**Table 3:** Associations between child ‘Oral’ and child ‘Social-Emotional’ sensory processing segments of the CSP2 and child appetitive traits at 9–11 years old (*n* = 130)

	Model 1						Model 2					
	B	95% CI		*R* ^2^	*p*-value		B	95% CI		Adj *R*^2^	*p*-value	
		Lower	Upper					Lower	Upper			
**Oral sensory processing**												
*Oral sensory processing (predictor)*												
Enjoyment of Food	-0.038	-0.053	-0.023	0.166	< 0.001	***	-0.038	-0.055	-0.022	0.203	< 0.001	***
*Oral sensory processing (predictor)*												
Desire to Drink	0.043	0.027	0.058	0.186	< 0.001	***	0.044	0.025	0.062	0.145	< 0.001	***
*Oral sensory processing (predictor)*												
Satiety Responsiveness	0.019	0.004	0.034	0.047	0.013	*	0.022	0.005	0.039	0.078	0.013	*
*Oral sensory processing (predictor)*												
Food Fussiness	0.077	0.059	0.095	0.351	< 0.001	***	0.080	0.059	0.101	0.351	< 0.001	***
**Social-Emotional responses associated with sensory processing**												
*Social-emotional (predictor)*												
Food Responsiveness	0.020	0.006	0.034	0.059	0.005	*	0.018	0.002	0.034	0.126	0.026	
*Social-emotional (predictor)*												
Emotional Overeating	0.015	0.006	0.025	0.072	0.002	**	0.013	0.002	0.024	0.096	0.022	
*Social-emotional (predictor)*												
Desire to Drink	0.025	0.012	0.038	0.102	< 0.001	***	0.023	0.008	0.038	0.041	0.004	**
*Social-emotional (predictor)*												
Emotional Undereating	0.015	0.004	0.027	0.049	0.011	*	0.018	0.004	0.031	0.035	0.011	*
*Social-emotional (predictor)*												
Food Fussiness	0.027	0.010	0.044	0.070	0.002	***	0.029	0.009	0.049	0.060	0.004	**

*Model 1 Unadjusted linear regression. Model 2 Multiple linear regression adjusted for child age at 9–11-year follow-up*,* child sex*,* breastfeeding exposure*,* whether complimentary feeding was introduced as per timing recommendations or not*,* maternal education level and original RCT allocation group*,* Maternal BMI at 9–11-year follow-up*,* Child BMI at 9–11-year follow-up.*

*The co-efficient for each CEBQ outcome is presented.*

*Social-Emotional = Social- Emotional responses associated with sensory processing.*

*CI Confidence Interval. B = Unstandardized coefficient.*

*R*^*2*^ *= variance explained within the linear regression model. Adjusted R*^*2*^ *= variance explained within the multiple regression model.*

Benjamini-Hochberg False Discovery Rate adjustment applied. *Q = 0.05, **Q = 0.01, ***Q = 0.001

### Correlation and multiple linear regression between maternal and child sensory profile quadrants

Examining relationships between maternal and child sensory profile quadrants, a positive relationship was observed between maternal ‘Sensory Sensitivity’ and child ‘Sensitivity/Sensor’ (*r* = 0.34), maternal ‘Sensation Avoiding’ and child ‘Avoiding/Avoider (*r* = 0.38) and maternal ‘Low Registration’ and child ‘Registration/Bystander’ (r = 0.47) (Additional file 6). In adjusted linear regression, a positive association was observed between maternal ‘Sensory Sensitivity’ and child ‘Sensitivity/Sensor’ (B = 0.345, CI = 0.133,0.574), maternal ‘Sensation Avoiding’ and child ‘Avoiding/Avoider’ (B = 0.550, CI = 0.285,0.814) and maternal ‘Low Registration’ and child ‘Registration/Bystander’ (B = 0.470, CI = 0.227,0.712) (Table 4).

**Table 4:** Associations between maternal and child sensory profile quadrants (*n* = 130)

	Model 1						Model 2					
	B	95% CI		*R* ^2^	*p*-value		B	95% CI		Adj *R*^2^	*p*-value	
		Lower	Upper					Lower	Upper			
**Quadrants**												
*Maternal Sensory Sensitivity (predictor)*												
Child Sensitivity/Sensor	0.347	0.136	0.558	0.077	0.001	**	0.354	0.133	0.574	0.127	0.002	**
*Maternal Sensation Avoiding (predictor)*												
Child Avoiding/Avoider	0.580	0.329	0.831	0.141	< 0.001	***	0.550	0.285	0.814	0.156	< 0.001	***
*Maternal Low Registration (predictor)*												
Child Registration/Bystander	0.521	0.295	0.748	0.140	< 0.001	***	0.470	0.227	0.712	0.147	< 0.001	***

*Model 1 Unadjusted linear regression; Model 2 Multiple linear regression adjusted for child age at 9–11-year follow-up*,* child sex*,* breastfeeding exposure*,* whether complimentary feeding was introduced as per timing recommendations or not and original RCT allocation group*,* Maternal BMI at 9–11-year follow-up*,* Child BMI at 9–11-year follow-up.*

*CI Confidence Interval.*

*B = Unstandardized coefficient; The co-efficient for the child’s sensory profile quadrants outcomes are presented.*

*R*^*2*^ *= variance explained within the linear regression model. Adjusted R*^*2*^ *= variance explained within the multiple regression model.*

Benjamini-Hochberg False Discovery Rate adjustment applied. *Q = 0.05, **Q = 0.01, ***Q = 0.001

## Discussion

Our results support our hypothesis that a child’s reaction or tolerance of oral sensory stimuli is related to child appetitive traits, specifically higher mean scores in ‘Food Fussiness’ and ‘Desire to Drink’ and lower mean scores in ‘Enjoyment of Food’. We observed positive associations between ‘Social-Emotional’ responses associated with sensory processing and ‘Desire to Drink’, ‘Emotional Undereating’ and ‘Food Fussiness’. We also observed that maternal and child sensory profiles were positively associated in three out of four sensory profile quadrants.

Child ‘Oral’ sensory processing was positively associated with ‘Food Fussiness’ even after adjusting for potential confounders. Statements within the construct of the ‘Oral’ sensory processing segment include *‘rejects certain tastes or food smells’*, and *‘gags easily from certain food textures’* indicate the crossover between sensory factors, such as smell, texture, color, and temperature and how they can relate to food refusal. A previous cross-sectional study of 73 children aged 2–5 years old, investigated associations between child oral sensory sensitivity and fruit and vegetable consumption. Results showed that children who were sensitive to taste/smell stimuli ate fewer fruit and vegetables, regardless of the influence of maternal intake [21]. Previous research has also shown that maternal fruit and vegetable intake is highly correlated their child’s intake [21, 56]. However, for children with oral sensory hypersensitivity, this heightened response to oral stimuli may negate the maternal modelling role due to strength of its impact, particularly with more challenging textures, smells or tastes. Our findings highlight the importance of considering oral sensory hypersensitivity in the typically developing older child that presents with persistent food fussiness.

Our results showed a negative association between child ‘Oral’ sensory processing and ‘Enjoyment of Food’. Among the numerous factors that contribute to eating and drinking, pleasure plays a key role. Food choices are driven by the positive neurological feedback that a food taste or texture provides [57]. This may be particularly relevant to children as other determining factors for food choice, such as the nutritional benefits of the food, or the importance of meeting dietary requirements will not be priorities [58]. Based on theoretical models addressing the determinants of food choice decision making, Marty, Chambaron [59] identified three main dimensions of pleasure derived from eating that are learned during childhood. These include, the sensory dimension of eating, which relates to the pleasure experienced before and after consuming food; the interpersonal dimension, which includes the social context of eating and the psychological dimension of eating, which relates to the cognitive pleasure of eating. In the optimal environment where healthy food choices are available and there is positive parental modelling, addressing sensory issues and therefore promoting a positive relationship with food may offer a strategy to avert or tackle fussy eating behavior.

An interesting finding in our study was that higher mean scores in ‘Oral’ sensory processing were positively associated with higher mean scores for ‘Desire to Drink’. The CEBQ item statements within the construct of ‘Desire to Drink’ do not specify the type of drinks the child is requesting or consuming, however in a study of 346 preschool children, higher scores in ‘Desire to Drink’ were associated with higher intakes of sugar sweetened beverages (SSBs) [60]. In clinical practice, children with excessive intakes of fluid, either sugar-sweetened or non-sweetened juice-based drinks often present with poor appetites and food fussiness. High intakes of fluids are often implicated as a reason for reduced appetite and refusal of meals. To date, research to confirm this assumption is lacking. In previous research of the current cohort, higher scores in ‘Desire to Drink’ were associated with lower scores in diet quality, at both 5 years (*n* = 306) and at 9–11 years old (*n* = 244) [61]. This suggests that high intakes of beverages may displace intake of foods that could contribute to a higher quality diet [61].

An explanation for the positive association observed between ‘Oral’ sensory processing and higher scores for ‘Desire to Drink’ could lie in the fact that the sensory properties of fluids may be less challenging texturally to a child with oral hypersensitivity. Fluid texture is generally consistent, which could promote acceptance as opposed to complex textures which are associated with food neophobia and fussy eating [62]. In previous cross-sectional research, a prevalent feeding issue identified in a cohort of 455 preschool children was preferring drinks to food [63]. Ross, Surette [64] identified the importance of including the question ‘*My child would rather drink than eat’* in their parental questionnaire developed to identify food texture sensitivity in children. In the current study we did not include information on the type and quantity of fluid the child was consuming in relation to ‘Desire to Drink’. Further research is required to explore relationships between children’s type and volume of fluid intake and how this relates to oral sensory sensitivity and heightened ‘Desire to Drink’, and ultimately how this may contribute to food fussiness.

There is limited research examining the relationship between a child’s social and emotional expressiveness and child appetitive traits. In our research a strong correlation was observed between the ‘Oral’ sensory processing segment of the CSP2 and the ‘Social-Emotional’ responses associated with sensory processing segment. Child ‘Social-Emotional’ responses were positively associated with ‘Desire to Drink’ and the food avoidant behaviors of ‘Emotional Undereating’ and ‘Food Fussiness’. With regard to ‘Food Fussiness’ and ‘Emotional Undereating’, previous work has demonstrated that individual differences in temperament may offer an explanation as to why some children develop food fussiness and others do not [42]. Children who have more emotional temperaments have been shown to display increased negativity at mealtimes and increased food avoidant eating behavior [42, 43, 65]. Being aware of a possible link between heightened ‘Social-Emotional’ responses associated with sensory processing and oral hypersensitivity may be helpful in understanding the sensory challenges that a child is encountering. It also may provide solutions on how to adapt the environment so that heightened sensory stimuli is reduced at mealtimes. However, research has also observed that children with higher emotionality and sensory reactivity are more likely to be perceived by their mothers as fussy eaters [43] and as our data was collected via maternal reported questionnaires, this may have biased our results.

The positive associations observed between ‘Social-Emotional’ responses and the food approach appetitive trait of ‘Desire to Drink’ may be reflective of the strong correlation between ‘Social Emotional’ responses to sensory processing and ‘Oral’ sensory processing observed in this cohort. Higher scores in both ‘Oral’ sensory processing and ‘Social Emotional’ responses were associated with higher mean scores in ‘Food Fussiness’ and ‘Desire to Drink’. As previous studies have observed that higher emotionality, anxiety and stress may result in higher consumption of food and beverages [66, 67], it was of interest that no relationship was observed between ‘Social-Emotional’ responses and ‘Emotional Overeating’. In this analysis we controlled for breastfeeding exposure and timing of introduction of complementary feeding as both have previously been demonstrated to impact child appetitive traits [35, 68]. However, we did not control for parental feeding practices. Responsive feeding practices encourage a child to self-regulate food and beverage intake. Non-responsive parental feeding practices often involve using food or drink in infancy and early childhood to placate heighted emotions [69]. This can disrupt a child’s self-regulation and promote the use of food to manage emotions. Furthermore, a higher emotional temperament in childhood has been linked with increased weight status and BMI z-score [70].

To our knowledge, the relationship between maternal sensory and child sensory profiles has not been explored to date. A small number of studies have examined associations between maternal eating behavior and child appetitive traits [35, 71]. Yelverton, Geraghty [35] observed a positive association between maternal ‘Emotional Eating’ and child ‘Emotional Over and Undereating’ at 5 years old (n = 230) [35]. Another study of 305 mother- child pairs (8–17 years old) observed that mothers and offspring shared similar disinhibited eating behaviours [71]. We observed a positive relationship between maternal and child ‘Sensory sensitivity’, ‘Avoidant’ and ‘Low registration’ sensory processing quadrants. As sensory processing patterns are thought to be inherent [2], these findings are not completely unexpected. However, an integral part of how a child learns to eat and drink is established through modelling the behaviors of parents and caregivers. Maternal ‘Sensation avoiding’ accounted for 16% of variance explained for the child ‘Avoiding/Avoider’ variable within the model. Our results pose the question as to what extent environmental factors, as opposed to genetic factors, influence a child’s sensory profile and whether a child’s sensory behaviors may mirror maternal behaviors. Further exploration is required to evaluate what proportion of sensory processing patterns are learned or shaped by the child’s role models.

Strengths of this study include the use of validated questionnaires to measure child appetitive traits and maternal and child sensory profiles. The availability of maternal information and demographics from the original ROLO pregnancy study, in combination with the 5-year-old and 9–11-year follow-up data, allowed for adjustment of important potential confounders in the analyses. We applied the Benjamini-Hochberg false discovery rate to adjust for multiple testing. The present study is not without limitations. This study is cross-sectional and exploratory in design, and therefore a power calculation was not performed. The questionnaires used to measure child appetitive traits and maternal and child sensory profiles were based on maternal self-report rather than direct observation of children’s appetitive traits and sensory processing patterns. Mothers may make assumptions about their children’s appetitive traits and sensory processing patterns. The use of the Short Sensory Profile 2 [72] which has been used in several other studies would have allowed better comparison with previous studies. However, use of the A/ASP and CSP2 allowed examination of associations between maternal and child sensory profile quadrants. Including data on the type and volume of drinks children were consuming in relation to the appetitive trait of ‘Desire to Drink’ within our analysis would have strengthened the interpretation of our findings. The majority of data were collected during the COVID-19 pandemic (2020–2022), although researchers instructed mothers to complete questionaries relating to their behviour pre-COVID-19, its influence may still have been present. While 432 participants returned for the ROLO Preteen follow-up, a much smaller number of participants completed both the CEBQ and the sensory profiles, therefore reducing our sample size. Finally, our sample may not be fully representative of the general population as the sample included a high proportion of mothers that had achieved tertiary or higher education level. In addition, as per the selection criteria for the original RCT, all children were second born children whose older sibling weighed over 4 kg at birth. This may have resulted in a cohort who are potentially obesogenic.

## Conclusion

Our findings highlight the need for increased awareness of the potential influence of oral sensory hypersensitivity and higher scores in ‘social-emotional’ sensory responses in typically developing children who presents with fussy eating. Identifying early modifiable factors that predispose fussy eating behavior could be valuable in providing direction on how to address sensitivity and ultimately improve dietary intake. The associations observed between both ‘Oral’ and ‘Social-Emotional’ sensory responses and ‘Desire to Drink’ require further investigation to elucidate how this may impact overall food intake. A deeper awareness and consideration of maternal sensory processing patterns and how they may influence their offspring’s sensory processing patterns could help provide a clearer understanding of their child’s eating behavior.

## Electronic Supplementary Material

Below is the link to the electronic supplementary material.

Supplementary Material 1

Supplementary Material 2

## Data Availability

The datasets used and analyzed during the current study are not publicly available in line with ethical approval but are available from the corresponding author on reasonable request.

## Abbreviations

ROLO: Randomized cOntrol trial of a LOw glycaemic index diet in pregnancy to prevent macrosomia
CEBQ: Childrens Eating Behaviour Questionnaire
A/ASP: Adolescent/Adult Sensory Profile
CSP2: Child Sensory Profile 2

## References

[1] Blissett J, Fogel A. <ArticleTitle Language=“En”>Intrinsic and extrinsic influences on children’s acceptance of new foods. Physiol Behav. 2013;121:89–95. 23458629 10.1016/j.physbeh.2013.02.013

[2] Dunn W. The Impact of Sensory Processing Abilities on the Daily Lives of Young Children and Their Families: A Conceptual Model. Infants Young Child. 1997;9(4):23–35.

[3] Dunn W. Child Sensory Profile 2: Users Mannual. USA: Bloomington, MN.: Psych Corp; 2014.

[4] Coulthard HP, Thakker DM. Enjoyment of Tactile Play Is Associated with Lower Food Neophobia in Preschool Children. J Acad Nutr Diet. 2015;115(7):1134–40. 25935569 10.1016/j.jand.2015.02.020

[5] Farrow CV, Coulthard H. Relationships between sensory sensitivity, anxiety and selective eating in children. Appetite. 2012;58(3):842–6. 22326881 10.1016/j.appet.2012.01.017

[6] Chistol LT, Bandini LG, Must A, Phillips S, Cermak SA, Curtin C. Sensory Sensitivity and Food Selectivity in Children with Autism Spectrum Disorder. J Autism Dev Disord. 2018;48(2):583–91. 29116421 10.1007/s10803-017-3340-9 PMC6215327

[7] Cermak SA, Curtin C, Bandini LG. Food Selectivity and Sensory Sensitivity in Children with Autism Spectrum Disorders. J Am Diet Assoc. 2010;110(2):238–46. 20102851 10.1016/j.jada.2009.10.032 PMC3601920

[8] Elsayed HE, Thompson KL, Conklin JL, Watson LR. Systematic Review of the Relation Between Feeding Problems and Sensory Processing in Children With Autism Spectrum Disorder. Am J Speech Lang Pathol. 2022;31(6):2875–99. 36356224 10.1044/2022_AJSLP-21-00401

[9] Leader G, Tuohy E, Chen JL, Mannion A, Gilroy SP. Feeding Problems, Gastrointestinal Symptoms, Challenging Behavior and Sensory Issues in Children and Adolescents with Autism Spectrum Disorder. J Autism Dev Disord. 2020;50(4):1401–10. 31955310 10.1007/s10803-019-04357-7

[10] Hubbard KL, Anderson SE, Curtin C, Must A, Bandini LG. A Comparison of Food Refusal Related to Characteristics of Food in Children with Autism Spectrum Disorder and Typically Developing Children. J Acad Nutr Diet. 2014;114(12):1981–7. 24928779 10.1016/j.jand.2014.04.017 PMC4252256

[11] Carnell S, Wardle J. Appetite and adiposity in children: evidence for a behavioral susceptibility theory of obesity. Am J Clin Nutr. 2008;88(1):22–9. 18614720 10.1093/ajcn/88.1.22

[12] Dovey JM, Staples PA, Gibson EL, Halford JC. Food neophobia and ‘picky/fussy’ eating in children: a review. Appetite. 2008;50(2–3):181–93. 17997196 10.1016/j.appet.2007.09.009

[13] Wolstenholme H, Kelly C, Hennessy M, Heary C. Childhood fussy/picky eating behaviours: a systematic review and synthesis of qualitative studies. Int J Behav Nutr Phys Act. 2020;17(1):2–22. 31900163 10.1186/s12966-019-0899-x PMC6942299

[14] Delahunt A, Killeen SL, O’Brien EC, Geraghty AA, O’Reilly SL, McDonnell CM, et al. Stability of child appetitive traits and association with diet quality at 5 years and 9–11 years old: Findings from the ROLO longitudinal birth cohort study. Eur J Clin Nutr. 2024;78(7):607–14. 38575724 10.1038/s41430-024-01436-6 PMC11230891

[15] van Dijk M, Bruinsma E, Hauser MP. The relation between child feeding problems as measured by parental report and mealtime behavior observation: A pilot study. Appetite. 2016;99:262–7. 26804043 10.1016/j.appet.2016.01.026

[16] Martins Y, Pliner P. Human food choices: An examination of the factors underlying acceptance/rejection of novel and familiar animal and nonanimal foods. Appetite. 2005;45(3):214–24. 16188344 10.1016/j.appet.2005.08.002

[17] Dunn W. The short sensory profile. USA: The Psychological Corporation; 1999.

[18] Cappellotto M, Olsen A. Food Texture Acceptance, Sensory Sensitivity, and Food Neophobia in Children and Their Parents. Foods. 2021;10(10):2327. 34681376 10.3390/foods10102327 PMC8535628

[19] Chow CY, Skouw S, Bech AC, Olsen A, Bredie WLP. A review on children’s oral texture perception and preferences in foods. Crit Rev Food Sci Nut. 2024;64(12):3861–79. 10.1080/10408398.2022.2136619 36300653

[20] Campos-Sánchez I, Muñoz-Sánchez R, Navarrete-Muñoz E-M, Molina-Iñigo MS, Hurtado-Pomares M, Fernández-Pires P, et al. Association between sensory reactivity and feeding problems in school-aged children: InProS Study. Appetite. 2024;192:107108. 10.1016/j.appet.2023.107108 39491151

[21] Coulthard H, Blissett J. Fruit and vegetable consumption in children and their mothers. Moderating effects of child sensory sensitivity. Appetite. 2009;52(2):410–5. 19110019 10.1016/j.appet.2008.11.015

[22] Liu X, Zhou Q, Clarke K, Younger KM, An M, Li Z, et al. Maternal feeding practices and toddlers’ fruit and vegetable consumption: results from the DIT-Coombe Hospital birth cohort in Ireland. Nutr J. 2021;20(1):1–84. 34666760 10.1186/s12937-021-00743-z PMC8524861

[23] Laureati M, Sandvik P, Almli L, Sandell V, Zeinstra M, Methven GG. Individual differences in texture preferences among European children: Development and validation of the Child Food Texture Preference Questionnaire (CFTPQ). Food Qual Prefer. 2020;80:103828.

[24] Dubois L, Farmer AP, Girard M, Peterson KE. Preschool children’s eating behaviours are related to dietary adequacy and body weight. Eur J Clin Nutr. 2007;61(7):846–55. 17180152 10.1038/sj.ejcn.1602586

[25] Dubois L, Farmer A, Girard M, Peterson K, Tatone-Tokuda F. Problem eating behaviors related to social factors and body weight in preschool children: A longitudinal study. Int J Behav Nutr Phys Act. 2007;4(1):9. 17408478 10.1186/1479-5868-4-9 PMC1855064

[26] Dubois L, Girard M, Potvin Kent M, Farmer A, Tatone-Tokuda F. Breakfast skipping is associated with differences in meal patterns, macronutrient intakes and overweight among pre-school children. Pub Health Nutr. 2009;12(1):19–28. 18346309 10.1017/S1368980008001894

[27] Ashcroft J, Semmler C, Carnell S, Van Jaarsveld CHM, Wardle J. Continuity and stability of eating behaviour traits in children. Eur J Clin Nutr. 2008;62(8):985–90. 17684526 10.1038/sj.ejcn.1602855

[28] Herle M, De Stavola B, Hubel C, Santos Ferreira DL, Abdulkadir M, Yilmaz Z, et al. Eating behavior trajectories in the first 10 years of life and their relationship with BMI. Int J Obes. 2020;44(8):1766–75. 10.1038/s41366-020-0581-z PMC7610465 32461555

[29] Taylor CM, Hays NP, Emmett PM. Diet at Age 10 and 13 Years in Children Identified as Picky Eaters at Age 3 Years and in Children Who Are Persistent Picky Eaters in A Longitudinal Birth Cohort Study. Nutrients. 2019;11(4):807. 30974806 10.3390/nu11040807 PMC6521015

[30] Taylor CM, Northstone K, Wernimont SM, Emmett PM. Macro- and micronutrient intakes in picky eaters: a cause for concern? Am J Clin Nutr. 2016;104(6):1647–56. 27935522 10.3945/ajcn.116.137356 PMC5118732

[31] Taylor CM, Emmett PM. Picky eating in children: causes and consequences. Proc Nutr Soc. 2019;78(2):161–9. 30392488 10.1017/S0029665118002586 PMC6398579

[32] Llewellyn CH, Van Jaarsveld CH, Johnson L, Camell S, Wardle J. Nature and nurture in infant appetite: analysis of the Gemini twin birth cohort. Am J Clin Nutr. 2010;91(5):1172–9. 20335548 10.3945/ajcn.2009.28868

[33] Fildes A, Jaarsveld CHMv, Cooke L, Wardle J, Llewellyn CH. Common genetic architecture underlying young children’s food fussiness and liking for vegetables and fruit. Am J Clin Nutr. 2016;103(4):1099–104. 26864359 10.3945/ajcn.115.122945 PMC4807704

[34] Dunn W. The Sensory Profile: Examiners Mannual. San Antonio, Texas, USA Pyschological Corporation. 1999.

[35] Yelverton CA, Geraghty AA, O Brien EC, Killeen S, Horan MC, Donnelly JM, et al. Breastfeeding and maternal eating behaviours are associated with child eating behaviours: findings from the ROLO Kids Study. Eur J Clin Nutr. 2021;75(4):670–9. 32999419 10.1038/s41430-020-00764-7 PMC8035071

[36] Gray HL, Buro AW, Sinha S. Associations Among Parents’ Eating Behaviors, Feeding Practices, and Children’s Eating Behaviors. Mat Child Health J. 2023;27(2):202–9. 10.1007/s10995-022-03572-6 36609937

[37] Schoeppe S, Vandelanotte C, Bere E, Lien N, Verloigne M, Kovács É, et al. The influence of parental modelling on children’s physical activity and screen time: Does it differ by gender? Eur J Pub Health. 2017;27(1):152–7. 28177458 10.1093/eurpub/ckw182

[38] Mascola AJ, Bryson SW, Agras WS. Picky eating during childhood: A longitudinal study to age 11 years. Eat Behav. 2010;11(4):253–7. 20850060 10.1016/j.eatbeh.2010.05.006 PMC2943861

[39] Zucker N, Copeland W, Franz L, Carpenter K, Keeling L, Angold A, et al. Psychological and Psychosocial Impairment in Preschoolers With Selective Eating. Pediatr (Evanston). 2015;136(3):e582–90. 10.1542/peds.2014-2386 PMC4552088 26240213

[40] Jacobi C, Agras WS, Bryson S, Hammer LD. Behavioral validation, precursors, and concomitants of picky eating in childhood. J Dev Behav Pediatr. 2003;24(3):211. 10.1097/00004583-200301000-00013 12500079

[41] Stifter CA, Moding KJ. Temperament in obesity-related research: Concepts, challenges, and considerations for future research. Appetite. 2019;141:104308. 31158396 10.1016/j.appet.2019.05.039 PMC8526117

[42] Haycraft E, Farrow C, Meyer C, Powell F, Blissett J. Relationships between temperament and eating behaviours in young children. Appetite. 2011;56(3):689–92. 21316412 10.1016/j.appet.2011.02.005

[43] Rendall S, Harvey K, Tavassoli T, Dodd H. Associations between emotionality, sensory reactivity and food fussiness in young children. Food Qual Pref. 2022;96:104420.

[44] Hafstad GS, Abebe DS, Torgersen L, von Soest T. Picky eating in preschool children: The predictive role of the child’s temperament and mother’s negative affectivity. Eat Behav. 2013;14(3):274–7. 23910765 10.1016/j.eatbeh.2013.04.001

[45] Walsh JM, McGowan CA, Mahony R, Foley ME, McAuliffe FM. Low glycaemic index diet in pregnancy to prevent macrosomia (ROLO study): randomised control trial. BMJ. 2012;345:e5605. 22936795 10.1136/bmj.e5605 PMC3431285

[46] O’Brien EC, Geraghty AA, McAuliffe FM. Successful strategies to improve follow-up for longitudinal birth cohort studies. Contemp Clin Trials. 2017;57:8–9. 28302569 10.1016/j.cct.2017.03.003

[47] Fewtrell M, Bronsky J, Campoy C, Domellöf M, Embleton N, Fidler Mis N, et al. Complementary Feeding: A Position Paper by the European Society for Paediatric Gastroenterology, Hepatology, and Nutrition (ESPGHAN) Committee on Nutrition. J Pediatr Gastro Nut. 2017;64(1):119–32. 10.1097/MPG.0000000000001454 28027215

[48] Health Service Executive. Weaning starting your baby on solid food Ireland. 2019. [Accessed 12/02/2024] https://www2.hse.ie/wellbeing/babies-and-children/weaning-eating/weaning/solid-foods/

[49] World Health Organization. Body Mass Index classification 2012 [Accessed 18/07/2024] http://apps.who.int/bmi/index.jsp?introPage=intro_3.html

[50] Cole P. LMS Growth- a Microsoft Excel add-in to assess growth references based on the LMS method. 2012.

[51] de Onis M, Onyango AW, Siyam A, Nishida C, Siekmann J. Development of a WHO growth reference for school-aged children and adolescents. Bull World Health Organ. 2007;85(9):660–7. 18026621 10.2471/BLT.07.043497 PMC2636412

[52] Brown C, Dunn W. Adolescent/Adult Sensory Profile Manual. San Antonio, TX: Psychological Corporation; 2002.

[53] Wardle J, Guthrie CA, Sanderson S, Rappoport L. Development of the Children’s Eating Behaviour Questiionaire. J Clin Child Psychol. 2001;42:963–70. 10.1111/1469-7610.00792 11693591

[54] Carnell S, Wardle J. Measuring behavioural susceptibility to obesity: Validation of the child eating behaviour questionnaire. Appetite. 2007;48(1):104–13. 16962207 10.1016/j.appet.2006.07.075

[55] Sleddens EFC, Kremers SPJ, Thijs C. The Children’s Eating Behaviour Questionnaire: factorial validity and association with Body Mass Index in Dutch children aged 6–7. Int J Behav Nutr Phys Act. 2008;5(1):49. 18937832 10.1186/1479-5868-5-49 PMC2612017

[56] Wardle J, Carnell S, Cooke L. Parental control over feeding and children’s fruit and vegetable intake: How are they related? J Am Diet Assoc. 2005;105(2):227–32. 15668680 10.1016/j.jada.2004.11.006

[57] Veldhuizen MG, Rudenga KJ, Small DM. In: Kringlebach MLK, editor. The pleasure of taste, flavor and food. C Berridge. Pleasures of the brain New York Oxford University; 2010. pp. 146–68.

[58] Albuquerque P, Brucks M, Campbell MC, Chan K, Maimaran M, McAlister AR, et al. Persuading Children: a Framework for Understanding Long-Lasting Influences on Children’s Food Choices. Customer Needs Solutions. 2017;5(1–2):38–50.

[59] Marty L, Chambaron S, Nicklaus S, Monnery-Patris S. Learned pleasure from eating: An opportunity to promote healthy eating in children? Appetite. 2018;120:265–74. 28890391 10.1016/j.appet.2017.09.006

[60] Sweetman C, Wardle J, Cooke L. Soft drinks and ‘desire to drink’ in preschoolers. Int J Behav Nutr Phys Act. 2008;5(1):60. 19055714 10.1186/1479-5868-5-60 PMC2612018

[61] Delahunt A, Killeen SL, O'Brien EC, Geraghty AA, O'Reilly SL, McDonnell CM, Cushion R, Mehegan J, McAuliffe FM. Stability of child appetitive traits and association with diet quality at 5 years and 9-11 years old: Findings from the ROLO longitudinal birth cohort study. Eur J Clin Nutr. 2024 Jul;78(7):607-614. 10.1038/s41430-024-01436-6 PMC11230891 38575724

[62] Coulthard H, Harris G, Emmett P. Delayed introduction of lumpy foods to children during the complementary feeding period affects child’s food acceptance and feeding at 7 years of age. Mat Child Nutr. 2009;5(1):75–85. 10.1111/j.1740-8709.2008.00153.x PMC6860515 19161546

[63] Wright CM, Parkinson KN, Shipton D, Drewett RF, Pediatrics. (Evanston). 2007;120(4):e1069–75. 10.1542/peds.2006-2961 17908727

[64] Ross CF, Surette VA, Bernhard CB, Smith-Simpson S, Lee J, Russell CG, et al. Development and application of specific questions to classify a child as food texture sensitive. J Texture Stud. 2022;53(1):3–17. 34435671 10.1111/jtxs.12627

[65] Farrow C, Blissett J. Maternal cognitions, psychopathologic symptoms, and infant temperament as predictors of early infant feeding problems: A longitudinal study. Int J Eat Disord. 2006;39(2):128–34. 16231348 10.1002/eat.20220

[66] Kininmonth A, Smith A, Carnell S, Steinsbekk S, Fildes A, Llewellyn C. The association between childhood adiposity and appetite assessed using the Child Eating Behavior Questionnaire and Baby Eating Behavior Questionnaire: A systematic review and meta-analysis. Obes Rev. 2021;22(5):e13169-n/a. 33554425 10.1111/obr.13169

[67] Lazarevich I, Irigoyen Camacho ME, Velázquez-Alva MdC, Zepeda Zepeda M. Relationship among obesity, depression, and emotional eating in young adults. Appetite. 2016;107:639–44. 27620648 10.1016/j.appet.2016.09.011

[68] Shim JEP, Kim JS, Mathai RAMSRD, The SKRT, Team SKR. Associations of Infant Feeding Practices and Picky Eating Behaviors of Preschool Children. J Am Diet Assoc. 2011;111(9):1363–8. 21872699 10.1016/j.jada.2011.06.410

[69] Bergmeier H, Skouteris H, Horwood S, Hooley M, Richardson B. Associations between child temperament, maternal feeding practices and child body mass index during the preschool years: a systematic review of the literature. Obes Rev. 2014;15(1):9–18. 23957249 10.1111/obr.12066

[70] Faith MS, Hittner JB. Infant temperament and eating style predict change in standardized weight status and obesity risk at 6 years of age. Int J Obes. 2010;34(10):1515–23. 10.1038/ijo.2010.156 20805827

[71] Zocca JM, Shomaker LB, Tanofsky-Kraff M, Columbo KM, Raciti GR, Brady SM, et al. Links between mothers’ and children’s disinhibited eating and children’s adiposity. Appetite. 2011;56(2):324–31. 21182882 10.1016/j.appet.2010.12.014 PMC3055954

[72] McIntosh D, Miller L, Shyu V, Dunn W. Development and validation of the short sensory profile. Sens Profile Man. 1999;61:59–73.

