# Supplementary material for: Sensory processing and child appetitive traits: findings from the ROLO longitudinal birth cohort study
Source: Nutr J. 2024 Nov 1;23:136. doi: 10.1186/s12937-024-01040-1 (PMC11529070; doi:10.1186/s12937-024-01040-1)
Supplement: Supplementary file 1 — Supplementary Material 1 [file 12937_2024_1040_MOESM1_ESM.docx]

| **Additional file 1: Maternal sensory profile quadrant categories** | | |
| --- | --- | --- |
|  | **n** | **%** |
| **Maternal low registration** |  |  |
| Much less than others | 7 | 5.3 |
| Less than others | 29 | 22.3 |
| Similar to others | 72 | 55.7 |
| More than others | 21 | 16.0 |
| Much more than others | 1 | 0.8 |
| **Maternal sensation seeking** |  |  |
| Much less than others | 4 | 3.1 |
| Less than others | 27 | 20.8 |
| Similar to others | 84 | 64.6 |
| More than others | 13 | 10.0 |
| Much more than others | 2 | 1.5 |
| **Maternal sensory sensitivity** |  |  |
| Much less than others | 2 | 1.5 |
| Less than others | 22 | 16.9 |
| Similar to others | 77 | 59.2 |
| More than others | 25 | 19.2 |
| Much more than others | 4 | 3.1 |
| **Maternal sensation avoiding** |  |  |
| Much less than others | 3 | 2.3 |
| Less than others | 27 | 20.8 |
| Similar to others | 78 | 60.0 |
| More than others | 19 | 14.6 |
| Much more than others | 2 | 1.5 |

| **Additional file 2: Child sensory profile quadrant categories** | | |
| --- | --- | --- |
|  | n | % |
| **Child Registration/Bystander** |  |  |
| Much less than others | 1 | 0.8 |
| Less than others | 5 | 3.8 |
| Similar to others | 113 | 86.9 |
| More than others | 10 | 7.7 |
| Much more than others | 1 | 0.8 |
| **Child Seeking/Seeker** |  |  |
| Much less than others | 1 | 0.8 |
| Less than others | 15 | 11.5 |
| Similar to others | 108 | 83.1 |
| More than others | 7 | 4.6 |
| Much more than others | - | - |
| **Child Sensitivity/Sensor** |  |  |
| Much less than others | 2 | 1.5 |
| Less than others | 3 | 2.3 |
| Similar to others | 112 | 86.2 |
| More than others | 8 | 6.2 |
| Much more than others | 5 | 3.8 |
| **Child Avoiding/Avoider** |  |  |
| Much less than others | 2 | 1.5 |
| Less than others | 8 | 6.2 |
| Similar to others | 105 | 80.8 |
| More than others | 11 | 8.5 |
| Much more than others | 4 | 3.1 |

| **Additional file 3: Child sensory processing segments** | | |
| --- | --- | --- |
|  | **n** | **%** |
| **Child Visual sensory processing** |  |  |
| Much less than others | 1 | 0.8 |
| Less than others | 29 | 22.3 |
| Similar to others | 89 | 68.5 |
| More than others | 9 | 6.9 |
| Much more than others | 2 | 1.5 |
| **Oral sensory processing** |  |  |
| Much less than others | 1 | 0.8 |
| Less than others | 5 | 3.8 |
| Similar to others | 106 | 81.5 |
| More than others | 13 | 10.0 |
| Much more than others | 5 | 3.8 |
| **Social-Emotional responses associated with sensory processing** | | |
| Much less than others | 1 | 0.8 |
| Less than others | 4 | 3.1 |
| Similar to others | 103 | 79.2 |
| More than others | 16 | 12.3 |
| Much more than others | 6 | 4.6 |

| **Additional file 4: Correlation between child Oral sensory processing and child Social-Emotional sensory processing at 9-11yrs old (n=130**) | | |
| --- | --- | --- |
|  | **r** |  |
| Child Social-Emotional | 0.493 | *** |
| *Spearman’s bivariate correlations*  *Child Social-Emotional = Social-Emotional Responses associated with Sensory Processing*  *Benjamini Hochberg False Discovery Rate applied *Q = 0.05, **Q = 0.01, ***Q = 0.001* | | |

| **Additional file 5: Correlations between child ‘Oral’ and ‘Social-Emotional’ sensory processing segments of the Children’s Sensory Profile 2 and child appetitive traits at 9-11 years old (n=130)** | | | | |
| --- | --- | --- | --- | --- |
|  | **Oral** | | **Social-Emotional** | |
|  | **r** |  | **r** |  |
| Food Responsiveness | 0.15 |  | 0.23 | * |
| Emotional Overeating | 0.14 |  | 0.28 | ** |
| Enjoyment of Food | -0.32 | *** | -0.19 |  |
| Desire to Drink | 0.44 | *** | 0.44 | *** |
| Satiety Responsiveness | 0.24 | * | 0.03 |  |
| Slowness Eating | 0.14 |  | -0.08 |  |
| Emotional Undereating | 0.13 |  | 0.24 | * |
| Food Fussiness | 0.53 | *** | 0.29 | *** |
| *Values generated from Spearman’s correlation statistic.*  *Benjamini-Hochberg False Discovery Rate adjustment applied. *Q = 0.05, **Q = 0.01, ***Q = 0.001* | | | | |

| **Additional file 6: Correlation between maternal and child sensory profile quadrants** | | |
| --- | --- | --- |
|  | **r** |  |
| Maternal Sensory Sensitivity | 0.340 | ** |
| Maternal Avoiding | 0.377 | *** |
| Maternal Low Registration | 0.469 | *** |
| Maternal Sensation Seeking | 0.106 |  |
| *Spearman’s bivariate correlations*  *Benjamini Hochberg False Discovery Rate applied *Q = 0.05, **Q = 0.01, ***Q = 0.001* | | |
